# Supplementary material for: Refining Reproduction Number Estimates to Account for Unobserved Generations of Infection in Emerging Epidemics
Source: Clin Infect Dis. 2022 Feb 17;75(1):e114–21. doi: 10.1093/cid/ciac138 (PMC9402635; doi:10.1093/cid/ciac138)
Supplement: ciac138_suppl_Supplementary_Material [file ciac138_suppl_supplementary_material.docx]

### **Supplementary Information: Refining reproduction number estimates to account for unobserved generations of infections in emerging epidemics**

Authors: Andrea Brizzi^1+^, Megan O’Driscoll^2,3+^, Ilaria Dorigatti^2^

Author Affiliations:
^1^Department of Mathematics, Imperial College London, London, United Kingdom

^2^MRC Centre for Global Infectious Disease Analysis and Jameel Institute, School of Public Health, Imperial College London, London, United Kingdom
^3^Department of Genetics, University of Cambridge, Cambridge, United Kingdom

^+^ Equal contribution

*Sensitivity analysis on the generation interval distribution*

We conducted a sensitivity analyses to assess the impact of the assumed length of the mean generation interval on the reproduction number estimates obtained with EpiEstim and our proposed adjustment. We used the COVID-19 data described in the main section and considered three different possible mean distributions with the same coefficient of variation set to 2. In Figure S1 the generation interval mean and standard deviation are set to 4 and 2 respectively, in Figure S2 the generation interval mean and standard deviation are set to 6 and 3 respectively and Figure S3 the generation interval mean and standard deviation are set to 8 and 4 respectively.


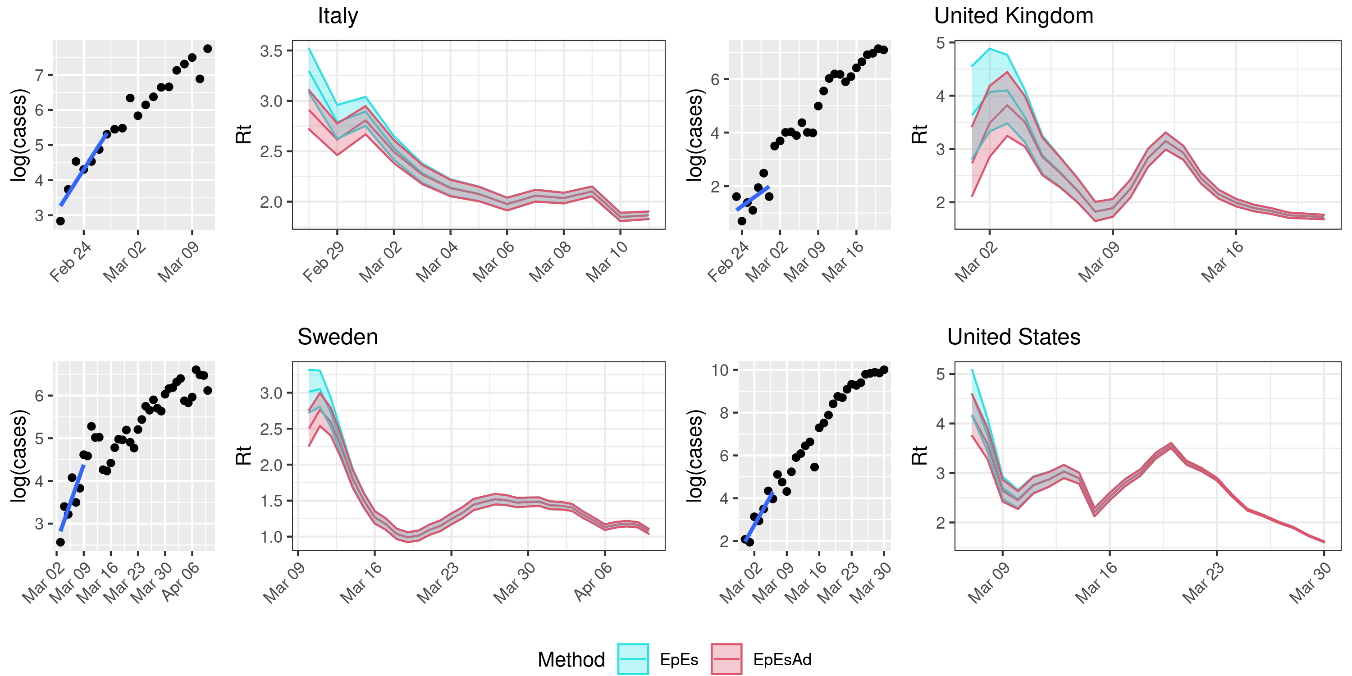


**Figure S1. Sensitivity analysis on generation interval**. Each quadrant includes a subfigure showing the logarithm of the data and the regression line (left) and the *R_t_* estimates obtained using a sliding window of 7 days (and the data up to that day) and a generation interval of mean 4 days and standard deviation of 2 days (right). Method abbreviations: EpiEstim (EpEs); Adjusted EpiEstim (EpEsAdj)

.
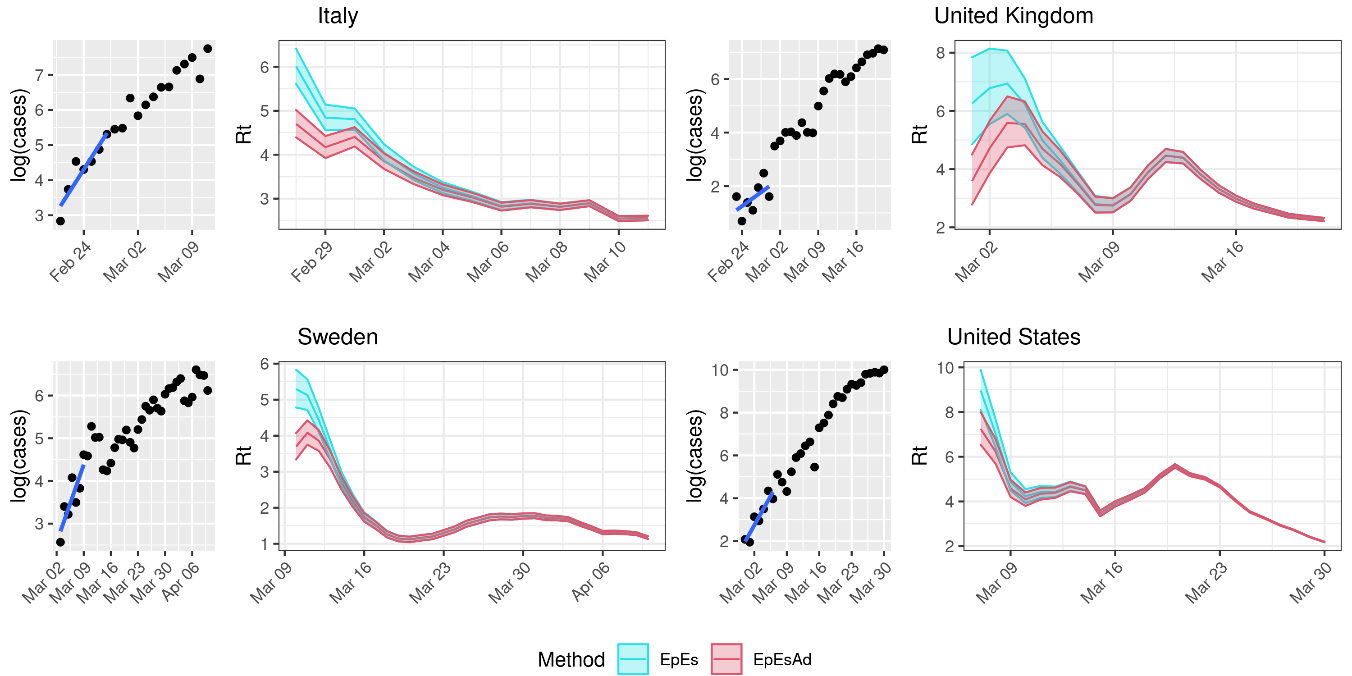


**S2. Sensitivity analysis on generation interval.** Each quadrant includes a subfigure showing the logarithm of the data and the regression line (left) and the *R_t_* estimates obtained using a sliding window of 7 days (and the data up to that day) and a generation interval of mean 6 days and standard deviation of 3 days (right). Method abbreviations: EpiEstim (EpEs); Adjusted EpiEstim (EpEsAdj).


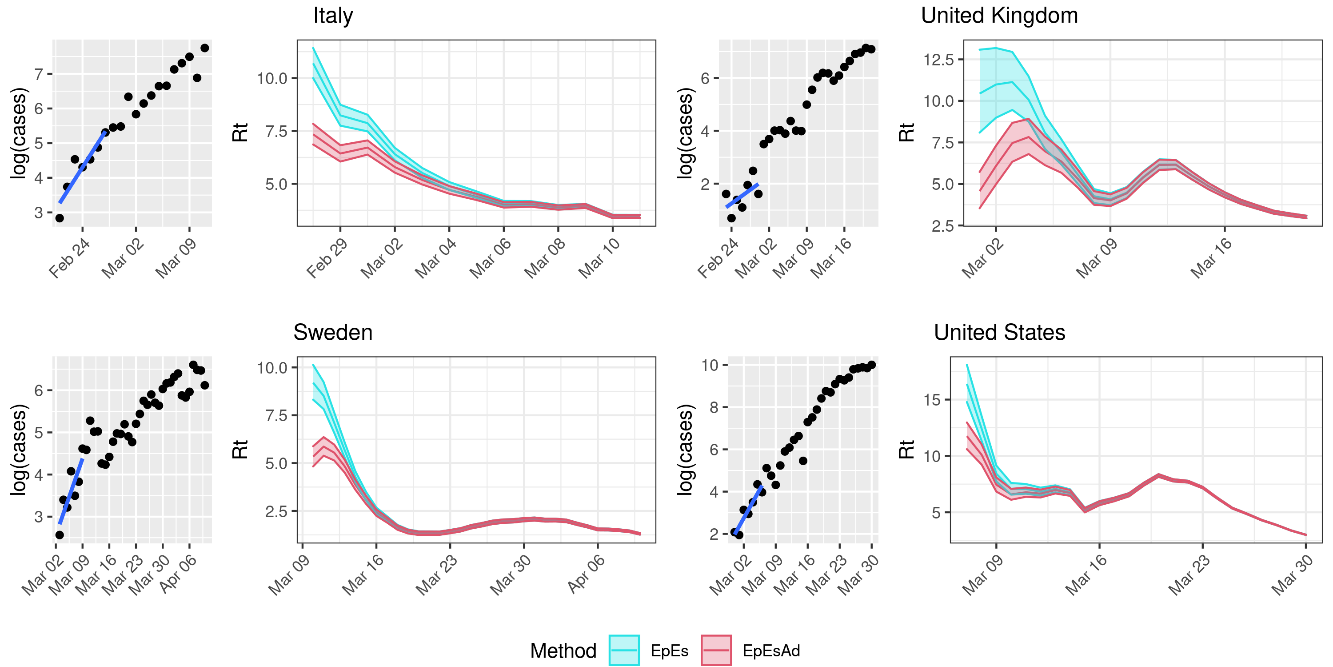


**Figure S3. Sensitivity analysis on generation interval.** Each quadrant includes a subfigure showing the logarithm of the data and the regression line (left) and the *R_t_* estimates obtained using a sliding window of 7 days (and the data up to that day) and a generation interval of mean 8 days and standard deviation of 4 days (right). Method abbreviations: EpiEstim (EpEs); Adjusted EpiEstim (EpEsAdj).

*Sensitivity analysis on the assumed reporting rate*

We also performed a sensitivity analysis to assess the impact of the assumed reporting rate on the *R_0_* estimates obtained in our simulations. The results are shown in Figures S4-S6.

Figure S4 considers a reporting rate set to 15%, Figure S5 considers a reporting rate set to 30%, while Figure S6 considers a reporting rate of 100%.


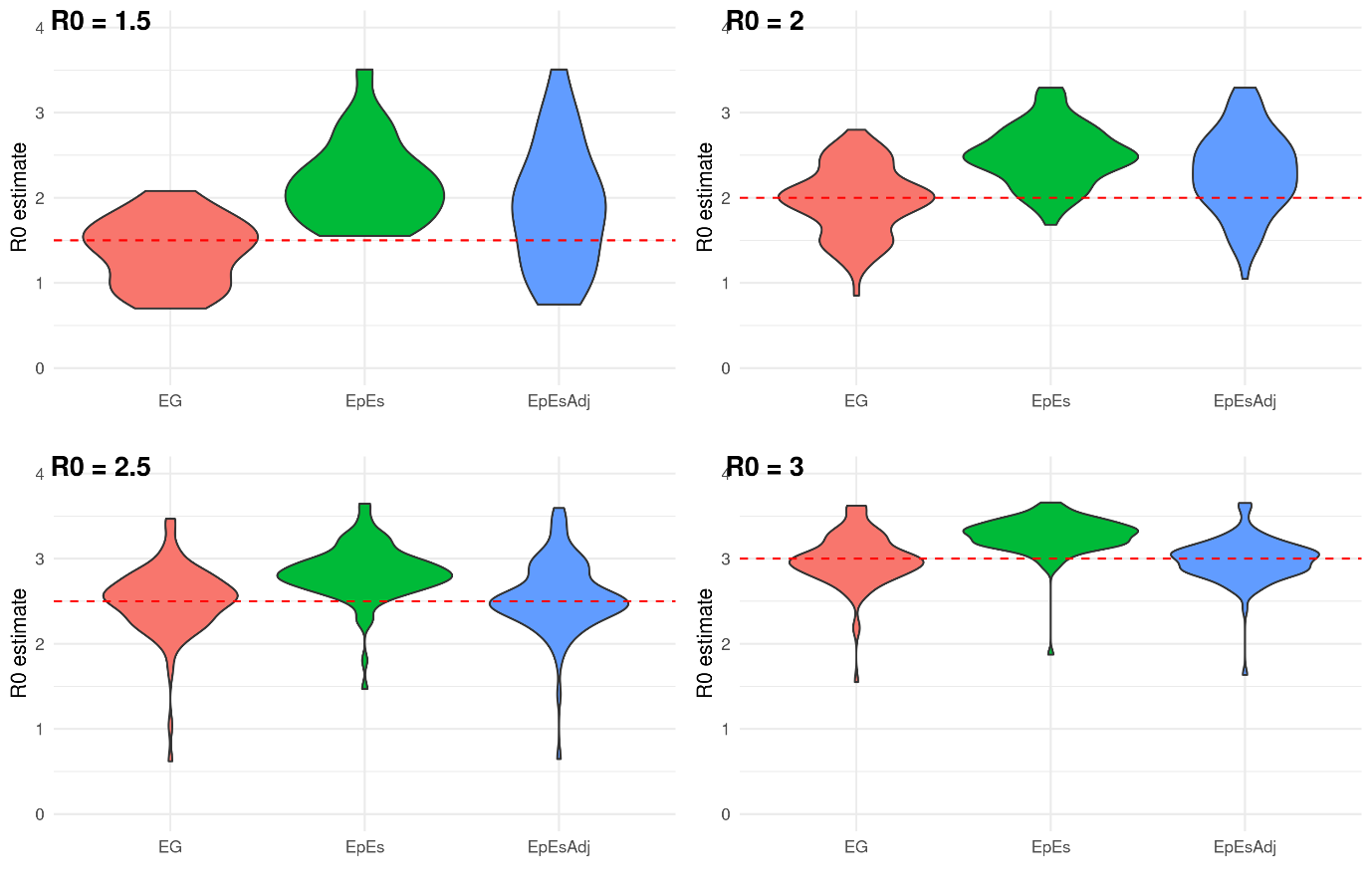
**Figure S4. Sensitivity analysis on reporting rates.** Distribution of mean R_0_ estimates assuming a fixed reporting rate ρ=15%. Each panel shows the distribution of the mean R_0_ estimates obtained using 100 simulations for a given true R_0_ value (red dashed line). Method abbreviations: Linear exponential growth rate method (EG); EpiEstim (EpEs); Adjusted EpiEstim (EpEsAdj).


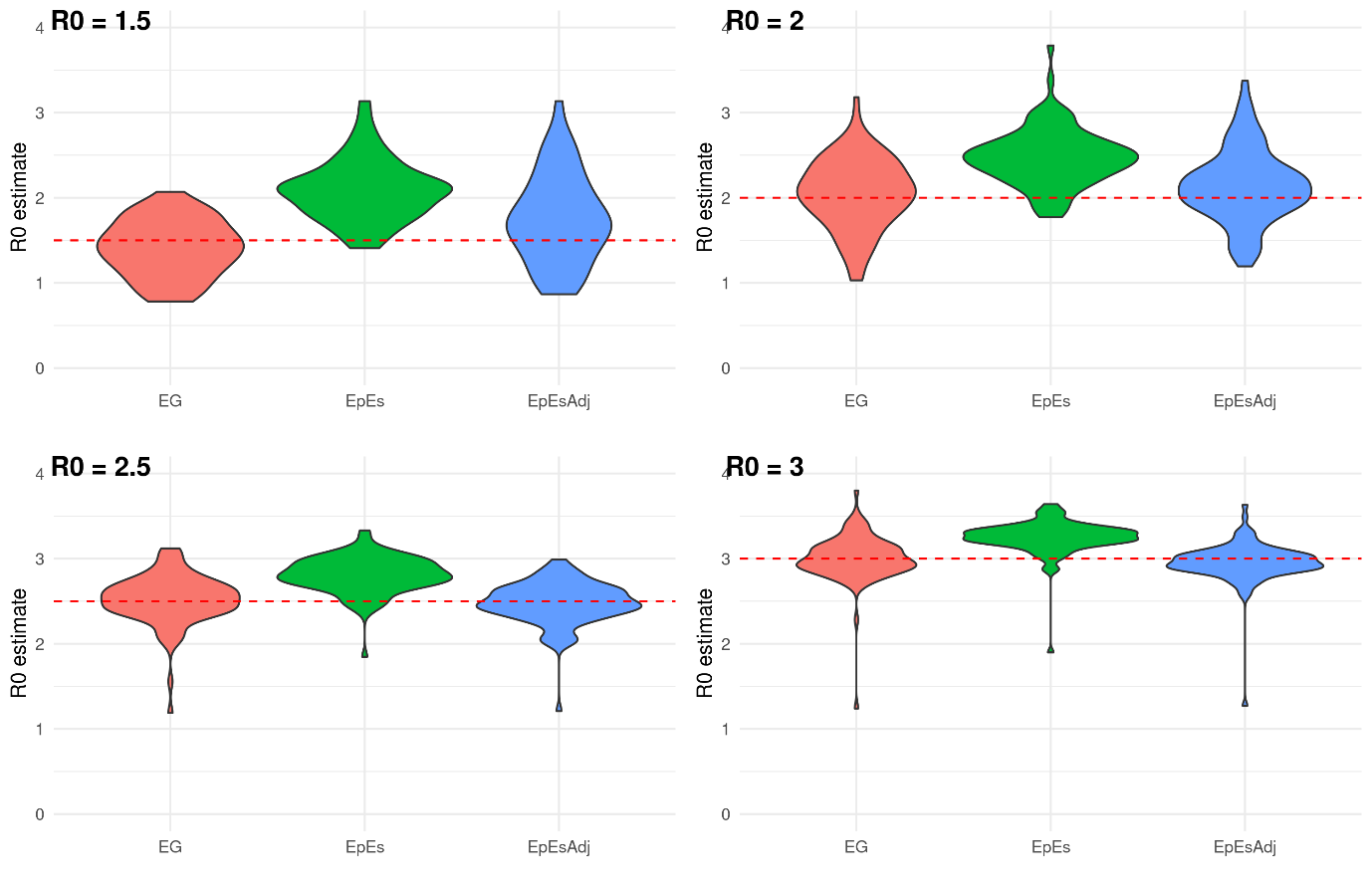
**Figure S5. Sensitivity analysis on reporting rates.** Distribution of mean R_0_ estimates assuming a fixed reporting rate ρ=30%. Each panel shows the distribution of the mean R_0_ estimates obtained using 100 simulations for a given true R_0_ value (red dashed line). Method abbreviations: Linear exponential growth rate method (EG); EpiEstim (EpEs); Adjusted EpiEstim (EpEsAdj).


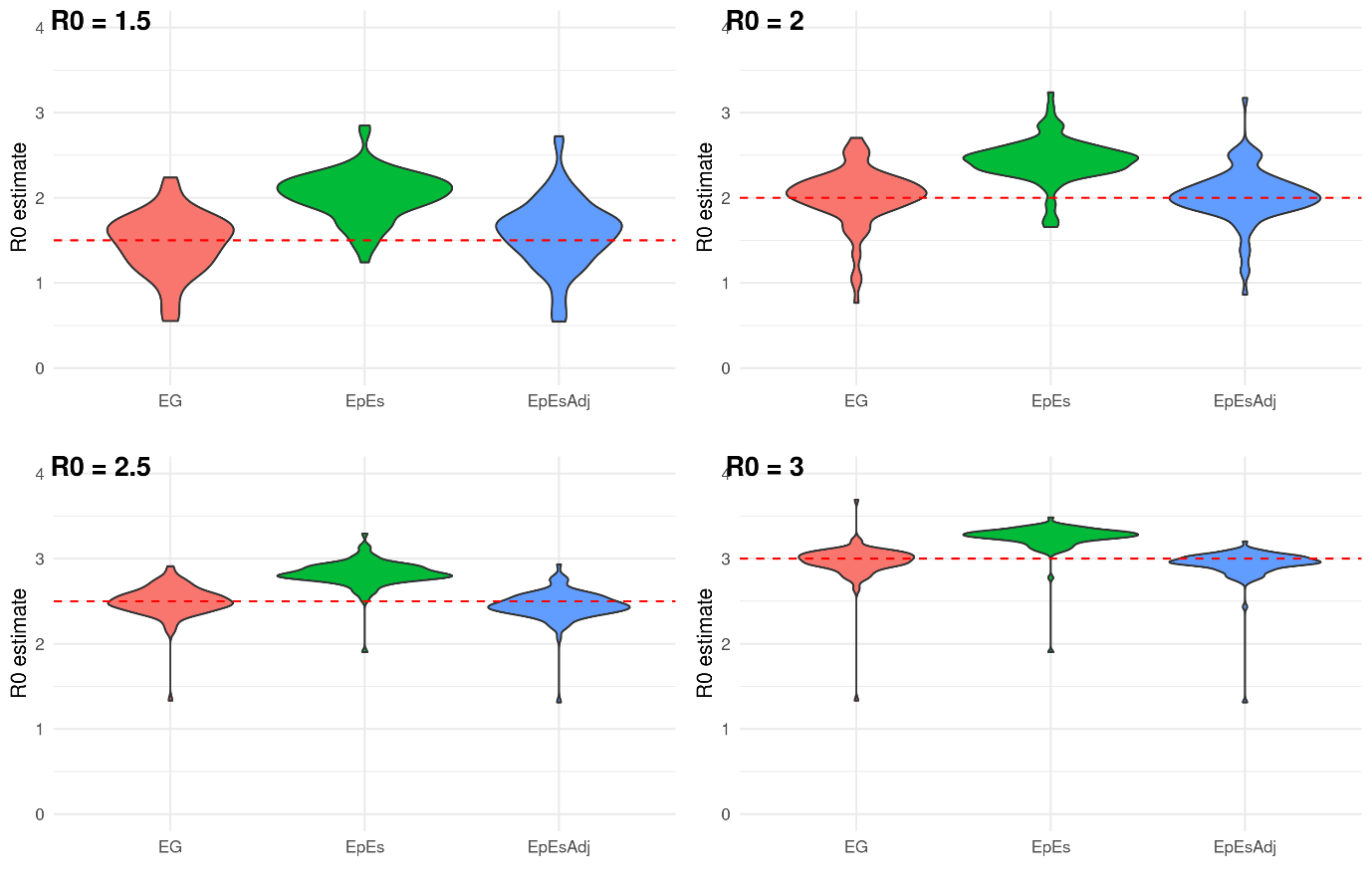
**Figure S6. Sensitivity analysis on reporting rates.** Distribution of mean R_0_ estimates assuming a fixed reporting rate ρ=100%. Each panel shows the distribution of the mean R_0_ estimates obtained using 100 simulations for a given true R_0_ value (red dashed line). Method abbreviations: Linear exponential growth rate method (EG); EpiEstim (EpEs); Adjusted EpiEstim (EpEsAdj).

*Sensitivity analysis on exponential growth assumption*

To assess the sensitivity of our adjustment to the exponential growth scenario, we further compared the performance of EpiEstim, EpiEstim adjusted and the Exponential Growth method on simulated data featuring sub-exponential growth dynamics.
Following Chowell et al. [1], we simulated an SEIR model with modified transition rates from the Susceptible to the Exposed compartment reproducing sub-exponential growth:

|  | $\frac{dS\left( t \right)}{dt}=-\beta S\left( t \right)\frac{{I\left( t \right)}^{\alpha}}{N},$ | ( 1a) |
| --- | --- | --- |
|  | $\frac{dE\left( t \right)}{dt}=\beta S\left( t \right)\frac{{I\left( t \right)}^{\alpha}}{N}-\gamma I\left( t \right),$ | ( 1b) |

Where $\alpha\in\left[ 0,1 \right]$ is a constant which quantifies the departure from the exponential growth scenario. Similarly to the main text scenario, we considered rates $\gamma=1/3$, $\sigma=1/3.5$. We further fixed $I\left( 0 \right)=5$, and $\beta=3\gamma$ and $N={10}^{6}$.
Note that equation (1a) and can be rewritten as $\frac{dS\left( t \right)}{dt}=-\beta'\frac{I\left( t \right)}{N}$, where $\beta^{'}$ corresponds to $\beta{I\left( t \right)}^{\alpha-1}$. For this reason, we can define the effective reproduction number as:

|  | $R_{t}=\frac{S\left( t \right)}{N}\frac{\beta}{\gamma}{I\left( t \right)}^{\alpha}.$ | (2) |
| --- | --- | --- |

In this scenario, the value of the basic reproduction number ($R_{0}$ evaluated at $t=0$), is now a function of the initial number of infections $I\left( 0 \right)$.

We run 200 simulations for each $\alpha$ value in $\left\{ 0.75,0.8,0.85,0.9,0.95,1 \right\}.$Figure S7 shows 5 randomly selected incidence curves for each value of $\alpha$.
In order to simulate missing generations of infections, we truncated the first 14 days of each incidence curve and run the methods to the successive 2 weeks of data. We then treated the first obtained estimate as $R_{0}$ estimate.

Figure S8 compares the evolution of the $R_{t}$ estimates obtained with EpiEstim and our adjustment on a randomly selected incidence curve simulated with $\alpha=0.7$ . As in the previous scenario, the proposed imputation of initial infections leads to smaller initial estimates, which then converge to the ones obtained with EpiEstim.
Figure S9 compares the distribution of the mean $R_{0}$ estimates obtained across the different $\alpha$ values and demonstrates that all methods based on the exponential growth assumption systematically underestimate the basic reproduction number, but the deviations get smaller as $\alpha$ approaches 1. This behaviour is to be expected: the concavity of the log of the incidence curves shown in Figure S7 suggests that the growth rate inferred in weeks 3 and 4 will be lower than the one that would be estimated in the first 2 weeks.

For smaller $\alpha$ values, we observe an apparent improved precision of the estimates obtained with EpiEstim compared with our adjustment (Figure S9), which is due to a trade-off between the underestimation described above with the over-estimation trend described in the main text. With larger values of $\alpha$, the under-estimation in $R_{t}$ plays a lesser role, yielding larger estimates of $R_{0}$ values obtained with EpiEstim and a better performance of our adjustment.

**Figure S7: Incidence curves simulated via a stochastic SEIR model with nonlinear incidence rates, N=10^6^, I(0)=5, γ=1/3, σ=1/3.5 and β=3γ.**For each value of $\alpha$ in $\left\{ 0.75,0.8,0.85,0.9,0.95,1 \right\}$, we show 5 randomly selected incidence curves. Days are indexed on the x-axis while the logarithm of the daily cases is reported on the y-axis.

**Figure S8. Visualisation of the adjustment method applied to simulated data following non-exponential growth.** On panel A, the logarithms of the first reported data (those in the green region) are used to fit a linear model (line). The linear model is then used to back-impute unobserved cases (red dots) to complement the available data (blue dots). On panel B, the true R_t_  value (black solid line) is compared to EpiEstim estimates without (blue) and with (red) adjustment using sliding windows of 7 days. The back-imputation reduces the initial mean estimates (dotted lines) and 95% credible interval widths (ribbons). The adjusted method then converges to the original method as the importance of the imputed datapoints vanishes. Method abbreviations: EpiEstim (EpEs); Adjusted EpiEstim (EpEsAdj)

**Figure S9. Distribution of mean R_0_ estimates obtained on simulated data following sub-exponential growth.**  Each panel shows the distribution of the mean R_0_ estimates obtained using 200 simulations for a given true R_0_ value (red dashed line). Method abbreviations: Linear exponential growth rate method (EG); EpiEstim (EpEs); Adjusted EpiEstim (EpEsAdj).

**References:**

1. Chowell G, Viboud C, Simonsen L, Moghadas SM. Characterizing the reproduction number of epidemics with early subexponential growth dynamics.
